# Genome-wide detection of short tandem repeat expansions by long-read sequencing

**DOI:** 10.1186/s12859-020-03876-w

**Published:** 2020-12-28

**Authors:** Qian Liu, Yao Tong, Kai Wang

**Affiliations:** 1 Raymond G. Perelman Center for Cellular and Molecular Therapeutics, Children’s Hospital of Philadelphia, Philadelphia, PA 19104 USA; 2 Department of Pathology and Laboratory Medicine, Perelman School of Medicine, University of Pennsylvania, Philadelphia, PA 19104 USA

**Keywords:** Short tandem repeats, Microsatellite, RepeatHMM, Repeat expansion, Repeat database

## Abstract

**Background:**

Short tandem repeat (STR), or “microsatellite”, is a tract of DNA in which a specific motif (typically < 10 base pairs) is repeated multiple times. STRs are abundant throughout the human genome, and specific repeat expansions may be associated with human diseases. Long-read sequencing coupled with bioinformatics tools enables the estimation of repeat counts for STRs. However, with the exception of a few well-known disease-relevant STRs, normal ranges of repeat counts for most STRs in human populations are not well known, preventing the prioritization of STRs that may be associated with human diseases.

**Results:**

In this study, we extend a computational tool RepeatHMM to infer normal ranges of 432,604 STRs using 21 long-read sequencing datasets on human genomes, and build a genomic-scale database called RepeatHMM-DB with normal repeat ranges for these STRs. Evaluation on 13 well-known repeats show that the inferred repeat ranges provide good estimation to repeat ranges reported in literature from population-scale studies. This database, together with a repeat expansion estimation tool such as RepeatHMM, enables genomic-scale scanning of repeat regions in newly sequenced genomes to identify disease-relevant repeat expansions. As a case study of using RepeatHMM-DB, we evaluate the CAG repeats of *ATXN3* for 20 patients with spinocerebellar ataxia type 3 (SCA3) and 5 unaffected individuals, and correctly classify each individual.

**Conclusions:**

In summary, RepeatHMM-DB can facilitate prioritization and identification of disease-relevant STRs from whole-genome long-read sequencing data on patients with undiagnosed diseases. RepeatHMM-DB is incorporated into RepeatHMM and is available at https://github.com/WGLab/RepeatHMM.

## Background

Short tandem repeat (STR) represents a consecutive repetition of a repeat motif with several (typically < 10) nucleotides. One example of STRs is a trinucleotide repeat, CTG*CTG*CTG*CTG*CTG*CTG*CTG with three nucleotides in the repeat unit (*i.e.*, CTG). Many STRs have variable repeat counts between different individuals. For example, CAG in the *ATXN3* gene may be repeated 14 times in one allele in a human genome, but 20 times in the other allele in the same human genome. Excessive repetition of specific STRs (i.e., repeat expansion) beyond normal ranges of repeat counts in control populations may lead to human diseases, such as Huntington’s diseases [1], spinocerebellar ataxia [2], fragile X syndrome [3], Friedreich’s ataxia [4], and others [5–7]. With the development and utilization of long-read sequencing techniques, more human diseases which are caused by repeat expansions have also been found in several recently published studies [8–11]. However, the normal ranges of different STRs may vary significantly. For example, repeats of CAG in *CACNA1A* in unaffected individuals typically range from 4 to 18 with a repeat count more than 21 considered as pathogenic, while repeats of CGG in *FMR1* in unaffected individuals typically range from 6 to 53. Thus, the knowledge of the normal repeat ranges of STRs is critically important to determine pathogenicity of observed repeats in known STRs or to discover novel disease-relevant repeat expansions, if repeat counts can be accurately quantified from long-read whole-genome sequencing data on a patient with undiagnosed diseases.

In existing studies, normal repeat range for a single STR is commonly inferred by experimental approaches on tens or more healthy individuals, and these experimental methods include capillary electrophoresis [12], gel electrophoresis [13], Southern blot analysis [14], electrochemical detection [15], melting curve analysis [16], mass spectrometry [17], and small-molecule biosensors [18]. However, it is too expensive and time/resource consuming to use these methods for determining normal repeat ranges for thousands of STRs at a genomic scale. As a result, we have knowledge on the normal ranges of repeat counts for only tens of well-studied repeats that are already known to cause diseases, which may delay the discovery of novel disease-causal repeats. Several recent studies have used short-read sequencing to infer repeat counts for STRs, such as lobSTR [19], RepeatSeq [20], STRviper [21], TREDPARSE [22], HipSTR [23], ExpansionHunter [24], and STRetch [25]; however, the intrinsic limitations of short-read sequencing prevent comprehensive characterization of all STRs or the discovery of novel disease-relevant repeat expansions that are longer than read length. Long-read sequencing techniques, such as PacBio sequencing and Oxford Nanopore sequencing, can be used to address these limitations; however, even when repeat counts for all STRs are determined, there is a general lack of reference databases to determine or prioritize which STRs have abnormally high number of repeats.

To overcome these limitations, in the current study, we design a framework to enable the discovery of abnormal repeat expansion of STRs from the increasing amounts of whole-genome long-read sequencing data. Long-read sequencing technologies produce reads longer than 10 kb which can span long STRs [26], and provide better coverage for longer repeat regions (> 300 bp). Thus, in this framework, we first extend RepeatHMM to enable the determination of repeat counts for genome-scale STRs, and name this module RepeatHMM-scan. Then, we use RepeatHMM-scan to detect repeat counts for all available STRs in human reference genome with 21 available long-read sequencing datasets, and summarize the results to build a reference database (RepeatHMM-DB) of normal repeat ranges of all annotated STRs in the human genome (with GRCh38 coordinates). After that, given a test long-read sequencing data, we use RepeatHMM or RepeatHMM-scan to determine repeat counts for an STR or a group of STRs, and compare the repeat counts against the corresponding STRs in RepeatHMM-DB to infer whether a repeat has excessive expansions outside normal repeat ranges. We stress here that in addition to RepeatHMM-scan, the RepeatHMM-DB can be also used in conjunction with other computational tools that infer repeat counts from long-read sequencing data. To demonstrate the usefulness of RepeatHMM-DB, (1) we compare the estimated repeat ranges against normal repeat ranges determined in existing works for 13 well-known repeats, and find that the inferred repeat ranges provide good estimation of repeat count ranges based on prior knowledge; (2) we compare the repeat counts of 5 unaffected individuals and 20 patients with Spinocerebellar ataxia type 3 (SCA3) against RepeatHMM-DB, and demonstrate the usefulness of the database in identifying pathogenic repeat expansions; and (3) we evaluate inferred repeat counts by RepeatHMM-scan from a test genome against RepeatHMM-DB, and find that our tool provides an efficient way for narrowing down candidate repeats from whole-genome repeats for de novo detection of pathogenic repeats. Thus, the RepeatHMM-DB database and the new RepeatHMM-scan module are expected to substantially facilitate analysis of STRs at a whole-genome scale. The new RepeatHMM-DB database and the RepeatHMM-scan module are incorporated into the latest version of RepeatHMM and are publicly accessible at https://github.com/WGLab/RepeatHMM.

## Methods

**Fig. 1:**
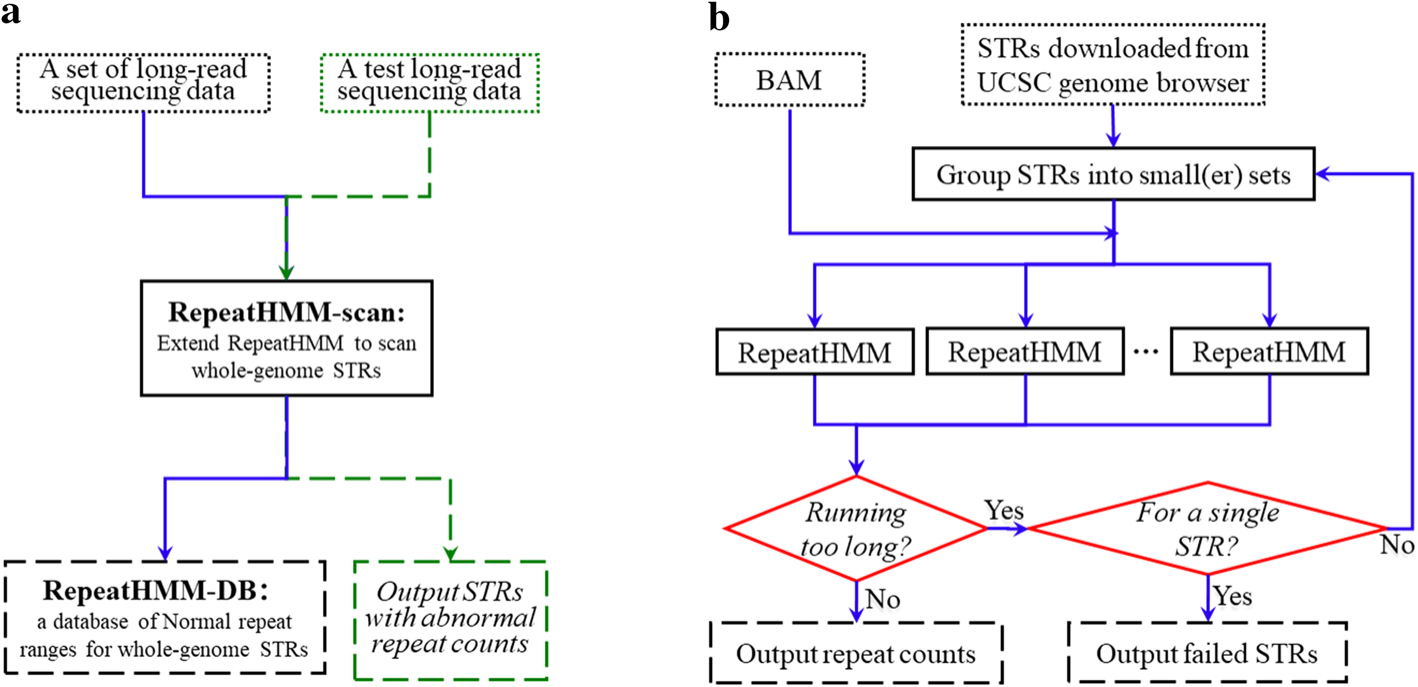
Overview of the computational framework. **a** The procedure for constructing RepeatHMM-DB. **b** The workflow of RepeatHMM-scan, which runs RepeatHMM on each STR set. Boxes in dots: *inputs*; Boxes in dashes: *outputs*; Lines and boxes in green: *testing process*; STR: short tandem repeats

The whole framework (Fig. 1a) proposed in this study has two main components: RepeatHMM-scan and RepeatHMM-DB. RepeatHMM-scan is extended from RepeatHMM [27]. RepeatHMM is an algorithm to estimate repeat counts from long-read sequencing data after taking high base calling error rate into consideration: it takes a set of reads (from which RepeatHMM generates a BAM file) or a BAM file as input, uses a split-and-align strategy to improve alignments, performs error correction, and leverages a hidden Markov model and Gaussian mixture model for peaking calling to infer repeat counts. Evaluation on both real SCA3 and SCA10 data sets [27] generated by the PacBio sequencer and various simulation data suggests the superior performance of RepeatHMM to infer repeat counts. Here, we extend RepeatHMM to achieve additional benefits from more and more available whole-genome long-read sequencing datasets.

### RepeatHMM-scan: scan whole-genome long-read sequencing data

It can be time-consuming to scan a long-read whole-genome sequencing data to determine repeat counts of tens of thousands of STRs. To speed up this step, we design a parallel scanning process called RepeatHMM-scan, which quickly determines repeat counts for all available STRs in a sequencing dataset. The framework of RepeatHMM-scan is shown in Fig. 1b.

**Fig. 2:**
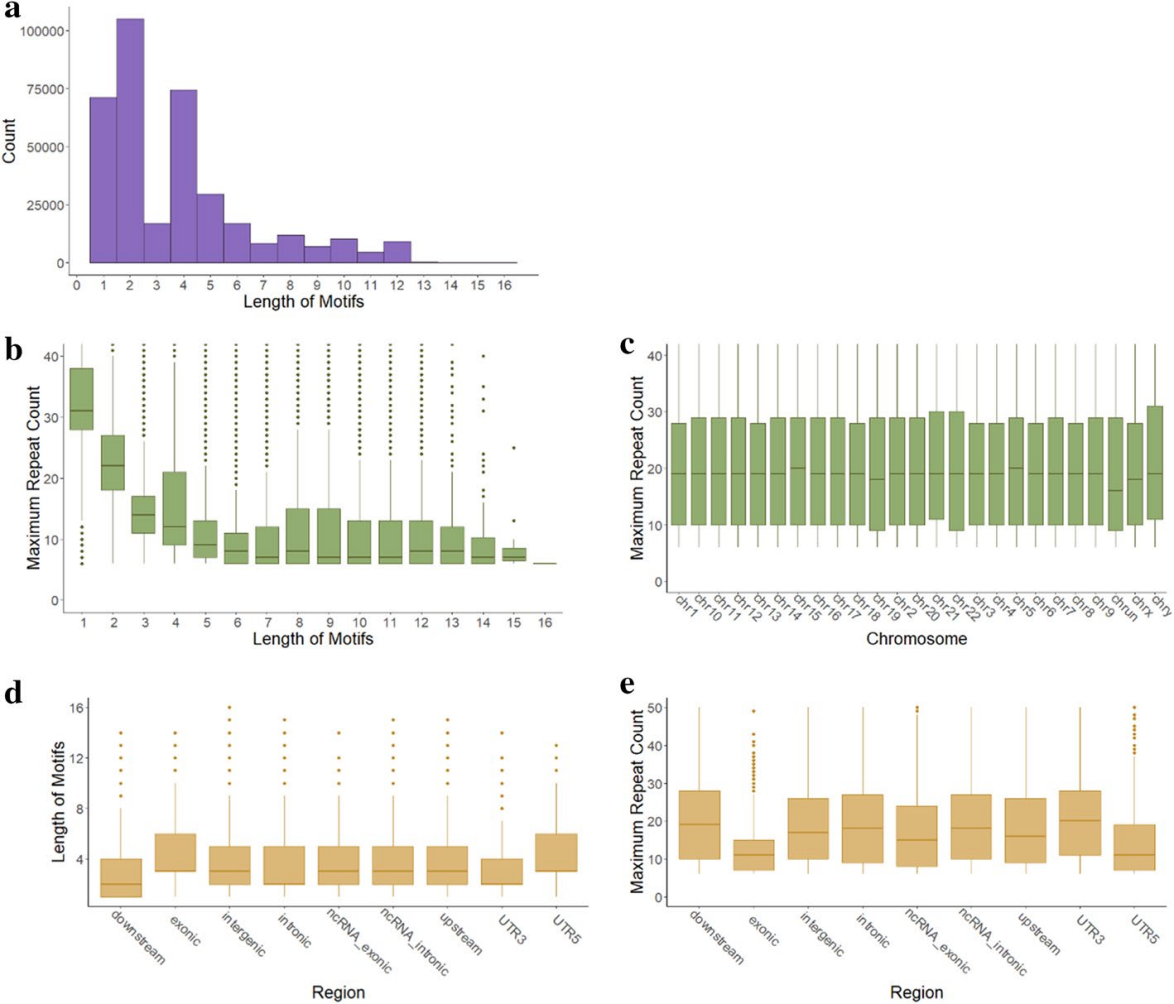
Whole genome STR analysis. **a** The numbers of STR with different motif sizes. **b** Distribution of the maximum repeat counts of STR repeats with different motif sizes. **c** Distribution of the maximum repeat counts along different chromosomes. STRs with large repeat counts (> 40) are not shown in **b** and **c**. Maximum repeat count is the maximum number of repeat occurrences among 21 long-read sequencing datasets for a motif at a locus. **d** The distribution of the motif sizes located in different regions in genomes. **e** The distribution of the maximum repeat counts of STR repeats located in different regions in genomes

The inputs of RepeatHMM-scan are a BAM file of a long-read whole-genome sequencing dataset and a set of STRs whose locus and repeat units are known. The information of those STRs is downloaded from UCSC genome browser [28] where tandem repeat finder [29] was used to detect all STRs in a reference genome. In this study, 432,604 STRs are downloaded from UCSC genome browser for the GRCh38/hg38 human reference genome. These STRs have different lengths of STR motifs. The distribution of these repeat motifs can be found in Fig. 2a where the length of the majority of repeat motifs ranges from 1 to 12, and dinucleotide and tetranucleotide repeats have more repeat instances.

Then, we use a divide-and-conquer strategy to group STRs into smaller sets, and run RepeatHMM on each set. We use a cluster system (i.e., Sun Grid Engine) to run multiple RepeatHMM jobs at the same time to speed up the process. But for some sets, the detection of an STR might take much more time to be done and might not be useful. We thus record running times for all finished RepeatHMM jobs, and kill those running jobs if they had been running for too long time (over a user-specified threshold). Then, STRs in the killed jobs are ignored or grouped into a much smaller set (based on users’ decision when scanning a new genome) on which a new RepeatHMM job runs until the final jobs only had one STR to be analyzed. Users can specify a threshold to filter the longest repeat regions to alter the job killing behavior. After that, repeat counts of all STRs whose repeat counts are successfully detected are combined in a single output file. Please note that this is an extended module in RepeatHMM, and takes the advantages of RepeatHMM to automatically scan a whole genome data for STR estimation and prioritization.

### RepeatHMM-DB

To build the first version of RepeatHMM-DB, we run RepeatHMM-scan on 21 long-read sequencing data for human genomes [30–35] (Table 1). Then, each repeat in the reference genome has 20 pairs (two genomes are haploid) of alleles of repeat counts. We assume the 21 sequencing datasets were generated from individuals unaffected by repeat expansion disorders, and then summarize the 40 repeat counts as normal repeat range for the repeats. After sorting the 40 repeat counts for a repeat locus, we obtain repeat counts at the minimum value and 95% percentile value to represent a robust normal repeat range so that maximum outliers are excluded. We summarize repeat counts for all STRs in a reference genome and build a database of normal repeat range for all available STRs, and name it RepeatHMM-DB.

**Table 1:** Whole-genome long-read sequencing datasets used in the current version of RepeatHMM-scan

Genome name	#Long reads	Mapped coverage
NA12878 [33]	68,064,542	54X
NA24385 [33]	26,325,971	55X
NA24149 [33]	12,927,769	26X
NA24143 [33]	12,655,875	26X
NA24631 [33]	20,640,162	56X
NA24694 [43]	10,211,241	28X
NA24695 [43]	10,075,227	28X
AK1 [32]	1,082,595,779	297X
CHM1 [35]	49,203,975	100X
CHM13 [44]	69,236,262	176X
HG00268 [34]	18,556,018	81X
HG00514 [34]	51,979,497	213X
HG00733 [34]	38,400,667	143X
HG01352 [34]	33,512,701	144X
HG02059 [34]	43,154,257	155X
HG02106 [34]	20,165,840	71X
HG02818 [34]	51,357,293	224X
HG04217 [34]	68,629,541	203X
NA19240 [34]	48,378,501	125X
NA19434 [34]	32,040,706	147X
HX1 [30]	27,541,832	84X

Reference assembly is GRCh38/hg38. All samples except HX1 were sequenced by the PacBio SMRT technology, while HX1 was sequenced by the Oxford Nanopore technology. CHM1 and CHM13 are haploid human genomes

Please note that RepeatHMM-DB is based on a data-driven construction benefiting from available sequencing data. As an increasing number of long-read sequencing datasets are available, RepeatHMM-DB could be significantly enhanced. Currently, RepeatHMM-DB does not document repeat ranges for pathogenic repeats. However, we provide options for users to improve RepeatHMM-DB by constructing repeat ranges of pathogenic or likely pathogenic STRs. There are several ways to achieve this goal. One is to use RepeatHMM to detect repeat counts of an STR on long-read data of patients with repeat expansion disorders, and the other is to manually construct pathogenic repeat ranges for those STRs if they can be compiled from existing studies in literature.

### Prioritization of STRs by RepeatHMM-DB

Given a test sequencing dataset, we use RepeatHMM or RepeatHMM-scan to determine repeat counts for a set of STRs of interest, and then compare those repeat counts with the corresponding normal repeat ranges in RepeatHMM-DB to see whether those STRs have much longer repeats. If so, the STR will be a good candidate for further investigation by experts or for functional annotation. If users already have the repeat count of a specific STR, they can simply extract normal repeat range information from RepeatHMM-DB to determine whether the STR has a repeat count within normal ranges.

### Long-read sequencing data for testing RepeatHMM-DB

To test whether the estimated normal repeat ranges are useful, we compare CAG repeat counts in the *ATXN3* gene (located on chromosome 14q [36, 37]) on 25 subjects against RepeatHMM-DB to infer the pathogenic status of the alleles and the subjects. These 25 subjects consisted of 20 patients affected with SCA3 [36, 37] and 5 control subjects: CAG repeat counts in the *ATXN3* gene for 20 patients were determined by capillary electrophoresis, and repeat counts for 5 control subjects were determined by Sanger sequencing. SCA3 is a rare autosomal dominant disease caused by abnormally extensive duplication of CAG repeats in the *ATXN3* gene. In healthy human subjects, the *ATXN3* gene usually contains 13 to 39 CAG repeats [38]. Extensive repeats with more than 55 CAG repeats in exons of *ATXN3* would affect pons and striatum, causing progressive cerebellar ataxia and even paralysis [39]. Amplicon sequencing data of CAG repeats in the *ATXN3* gene on 25 subjects was generated using the PacBio Sequel sequencer as previously published [27]. In the test, we use normal repeat ranges in RepeatHMM-DB to infer whether the subjects had SCA3 and do not use any prior normal and pathogenic repeat range knowledge in existing studies.

## Results

### Overview

In this study, based on the previous development of RepeatHMM, we design RepeatHMM-scan for the detection of repeat counts for whole-genome STRs. After running RepeatHMM-scan on 21 long-read sequencing data for human genomes, we build RepeatHMM-DB for normal repeat ranges for genome-wide STRs. Based on RepeatHMM-DB, we define a score to indicate whether a repeat count in a specific locus is abnormally high. Below, we discuss the utility of RepeatHMM-DB by comparing normal repeat ranges in RepeatHMM-DB for 13 well-known repeats, and by testing RepeatHMM-DB on the *ATXN3* gene to infer pathogenic repeats. We also demonstrate how to use RepeatHMM-DB for filtering whole-genome STRs for de novo detection of abnormal repeats that may be disease relevant.

### Inference of the ranges of the repeat counts for whole-genome STRs

We use RepeatHMM-scan in Fig. 1 to quantify the repeat counts for all STRs in the human genome on 21 long-read sequencing data sets for human genomes (Table 1), and summarize the repeat counts to construct RepeatHMM-DB. In RepeatHMM-DB, each row is for a repeat with several fields: the chromosome, the starting position of the repeat, the end position of the repeat, the minimum and 95% percentile of the repeat counts, the repeat counts in 21 available long-read sequencing data.

Right now, RepeatHMM-DB contains 432,604 STRs on the GRCh38/hg38 coordinate in total. The upper bounds of their normal repeat counts vary greatly. ~ 96% of maximum normal repeat counts are less than 60, but there are 5920 patterns that repeat more than 100 times. The lengths of the effective repeat patterns range from 1 to 16 bp (Fig. 2b). In general, average repeat counts decrease as repeat units get longer. Repeats located in most chromosomes have similar average counts, although some chromosomes (chr5 and chr15) have slightly higher average repeat counts and some are lower (chr19 and chrX) (Fig. 2c). We further run ANNOVAR [40] to obtain the locations of repeat loci, and we find there are 1060, 235,278, 144,912, 2716, 591, 24,379, 3190, 2058 and 714 repeat loci in exonic, intergenic, intronic, downstream, ncRNA_exonic, ncRNA_intronic, upstream, UTR3 and UTR5 regions, respectively. We then illustrate the distribution of motif sizes and of repeat counts in Fig. 2d, e. According to the box plots in Fig. 2, the differences of the length of motifs for repeat loci among different regions are significant, while repeat counts in exonic regions are usually smaller than those in intergenic and intronic regions, indicating that exonic regions are less tolerated with longer repeats than other regions.

**Fig. 3:**
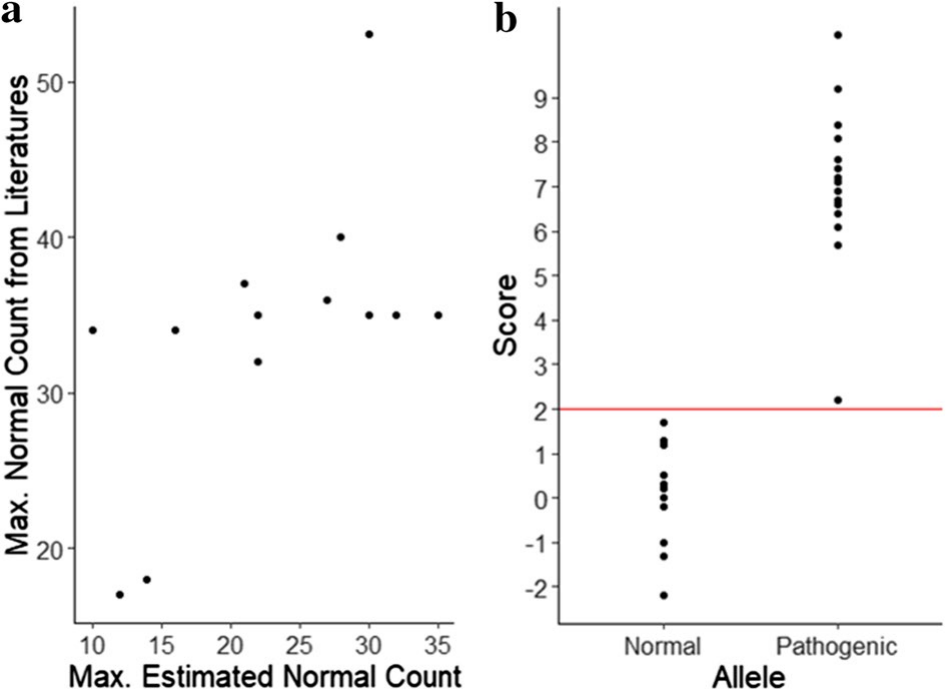
Inferred maximum normal repeat counts and its application on the ATXN3 data. **a** Comparison of inferred maximum normal counts of 13 genes with the maximum normal counts in prior knowledge from published literature. **b** Comparison of the scores of pathogenic alleles against normal alleles of ATXN3 genes in 20 patients with SCA3 and 5 control subjects. The red line represents a Z-score of 2

To check the accuracy of the normal ranges of STR repeats in RepeatHMM-DB, we compare the normal repeat ranges of 13 well-known disease-causal trinucleotide repeats in RepeatHMM-DB against prior knowledge extracted from existing literature, as shown in Table 2. It can be seen that RepeatHMM-DB closely resembles prior knowledge and provides a much richer set of information on the distribution of repeat counts for these well-studied STRs (Fig. 3a). Please note that (1) RepeatHMM-DB is constructed from 21 long-read sequencing datasets for human genomes, and it can be improved in the future when more long-read whole-genome sequencing datasets are available, (2) RepeatHMM-DB provides normal repeat ranges of many other repeats which are not available before (not shown in Table 2 but available together with the tool), and thus could facilitate the discovery of novel repeat expansion in STRs that are associated with human disorders, and (3) the sequencing data were not generated for the purpose of constructing RepeatHMM-DB, and we extract the population-wide repeat count information through re-using existing data.

**Table 2:** The normal repeat ranges for 13 genes estimated by RepeatHMM-DB, in comparison to normal/pathogenic repeat ranges based on prior knowledge from literature

Type	Gene	Codon	Normal	Est.	Ref.	Pathogenic
DRPLA (Dentatorubral-pallidoluysian atrophy)	ATN1 or DRPLA	CAG	6–35	11–22	19	49–88
HD (Huntington's disease)	HTT	CAG	6–35	17–30	21	36–250
SBMA (Spinal-bulbar muscular atrophy)	AR	CAG	9–36	18–27	23	38–62
SCA1 (Spinocerebellar ataxia Type 1)	ATXN1	CAG	6–35	23–32	29	49–88
SCA2 (Spinocerebellar ataxia Type 2)	ATXN2	CAG	14–32	15–22	23	33–77
SCA3 (Spinocerebellar ataxia Type 3)	ATXN3	CAG	12–39	11–28	14	55–86
SCA6 (Spinocerebellar ataxia Type 6)	CACNA1A	CAG	4–18	7–14	13	21–30
SCA7 (Spinocerebellar ataxia Type 7)	ATXN7	CAG	7–17	9–12	10	38–120
FRAXA (Fragile X syndrome)	FMR1, on the X-chr	CGG	6–53	20–30	20	230+/55–200
FRAXE (Fragile XE mental retardation)	AFF2, on the X-chr	CCG	6–35	25–35	29	200+
FRDA (Friedreich's ataxia)	FXN or X25	GAA	7–34	7–10	6	100+
DM (Myotonic dystrophy)	DMPK	CTG	5–34	5–16	20	50+
SCA8 (Spinocerebellar ataxia Type 8)	ATXN8 or SCA8	CTG	16–37	8–21	26	110–250

Est.: the estimated normal repeat ranges in RepeatHMM-DB; Ref.: the repeat counts from the reference genome GRCh38/hg38; Normal: the normal repeat ranges from prior knowledge

### Prediction of disease status using RepeatHMM-DB

We further test RepeatHMM-DB by using its normal range of CAG repeat of the *ATXN3* gene in RepeatHMM-DB to infer pathogenic alleles. The amplicon-based long-read sequencing data were sequenced for the *ATXN3* genes on 20 patients with SCA3 and 5 unaffected subjects, with their CAG repeat counts determined by capillary electrophoresis or Sanger sequencing techniques (Table 3). The amplicon data set was previously published to evaluate RepeatHMM on SCA3 [27]. We use the normal range of CAG repeat in *ATXN3* to determine which alleles are pathogenic or which patients have SCA3 by comparing the wet-lab determined repeat counts with the normal repeat range in *ATXN3* in RepeatHMM-DB. The results are shown in Table 3. In Table 2, RepeatHMM-DB suggests the normal repeats of *ATXN3* between 11 and 28 with a standard deviation 5.9. The deviation is calculated using $\sqrt {\sum\nolimits_{i}^{N} {(c_{i} - E_{c} )^{2} } {/}(N - 1)}$ where $c_{i}$ is a repeat count in $N$ repeat counts and $E$ is the mean of the $N$ repeat counts; the deviation thus is an estimation of how repeat counts for an STR are deviated from each other. Given a repeat count $c$, we use $\left( {c - 28} \right)/5.9$ to calculate a score to show how this repeat count is different from the maximum repeat count in RepeatHMM-DB for this STR. If this score is larger than a threshold, we consider this repeat count to be a pathogenic allele (Fig. 3b); otherwise, a normal allele. With a threshold of the score > 2.0, we can have the results in Table 3. Please note that the threshold of 2.0 is used due to that the CAG repeat is located in the coding region of *ATXN3*. The score threshold for non-coding STRs should be larger.

**Table 3:** The estimation of the normal/abnormal repeat status of CAG repeats in ATXN3 according to RepeatHMM-DB compared with the determination by prior literature knowledge of normal repeat range

*Subject*	True counts	Prior knowledge		RepeatHMM-DB		
		Allele	Subject	Score	Allele	Subject
SRR5363334	14, 77	N, P	P	− 2.2, 8.4	N, P	P
SRR5363452	30, 66	N, P	P	0.5, 6.6	N, P	P
SRR5363453	14, 69	N, P	P	− 2.2, 7.1	N, P	P
SRR5363454	14, 71	N, P	P	− 2.2, 7.4	N, P	P
SRR5363455	21, 72	N, P	P	− 1.0, 7.6	N, P	P
SRR5363456	14, 77	N, P	P	− 2.2, 8.4	N, P	P
SRR5363457	26, 71	N, P	P	− 0.2, 7.4	N, P	P
SRR5363458	14, 63	N, P	P	− 2.2, 6.1	N, P	P
SRR5363459	29, 70	N, P	P	0.3, 7.2	N, P	P
SRR5363460	27, 71	N, P	P	0.0, 7.4	N, P	P
SRR5363461	34, 75	N, P	P	1.2, 8.1	N, P	P
SRR5363462	28, 89	N, P	P	0.2, 10.4	N, P	P
SRR5363463	61, 61	P, P	P	5.7, 5.7	P, P	P
SRR5363464	26, 65	N, P	P	− 0.2, 6.4	N, P	P
SRR5363465	14, 89	N, P	P	− 2.2, 10.4	N, P	P
SRR5363466	40, 67	P, P	P	2.2, 6.7	P, P	P
SRR5363467	37, 68	N, P	P	1.7, 6.9	N, P	P
SRR5363468	28, 40	N, P	P	0.2, 2.2	N, P	P
SRR5363469	14, 82	N, P	P	− 2.2, 9.2	N, P	P
SRR5363470	14, 68	N, P	P	− 2.2, 6.9	N, P	P
SRR5363471	14, 14	N, N	N	− 2.2, − 2.2	N, N	N
SRR5363472	27, 35	N, N	N	0.0, 1.3	N, N	N
SRR5363473	28, 28	N, N	N	0.2, 0.2	N, N	N
SRR5363480	14, 14	N, N	N	− 2.2, − 2.2	N, N	N
SRR5363632	14, 19	N, N	N	− 2.2, − 1.3	N, N	N

The true repeat counts for 20 patients with SCA3 and 5 control subjects were determined by capillary electrophoresis or Sanger sequencing. The estimated normal repeat range of CAG repeat is 11–28. The ‘Allele’ column shows the pathogenic inference for each allele, while the ‘Subject” column shows the disease status prediction for each subject. ‘N’: normal, ‘P’: pathogenic/patient

Based on the inference in Table 3, we summarize the performance of inferring the pathogenic status of alleles. In the test data, the 25 subjects have 50 alleles (two alleles in a subject may have the same repeat counts if they are homozygous). With the score threshold > 2.0, all 22 pathogenic and 28 normal alleles are correctly classified, indicating that our tool can detect pathogenic allele as accurate as prior knowledge obtained by expensive and extensive wet-lab studies.

We then summarize the performance of identifying whether an individual is affected by SCA3. Each subject has 2 *ATXN3* alleles in autosomes, and if one of them is pathogenic, the subject is affected by SCA3 as it is an autosomal dominant disease. With the inference in Table 3, none of 20 SCA3 patients are misclassified as unaffected, and none of the unaffected subjects is incorrectly classified as being affected. This analysis demonstrates a perfect performance when using RepeatHMM-DB to detect affected patients without prior knowledge. Although this long-read dataset clearly shows that RepeatHMM-DB is able to precisely identify the pathogenic status of individuals, we also stress that it represents a specific case study and the approach requires further evaluation in future large-scale studies.

### A demonstration on the use of RepeatHMM-DB for whole-genome STR analysis

**Fig. 4:**
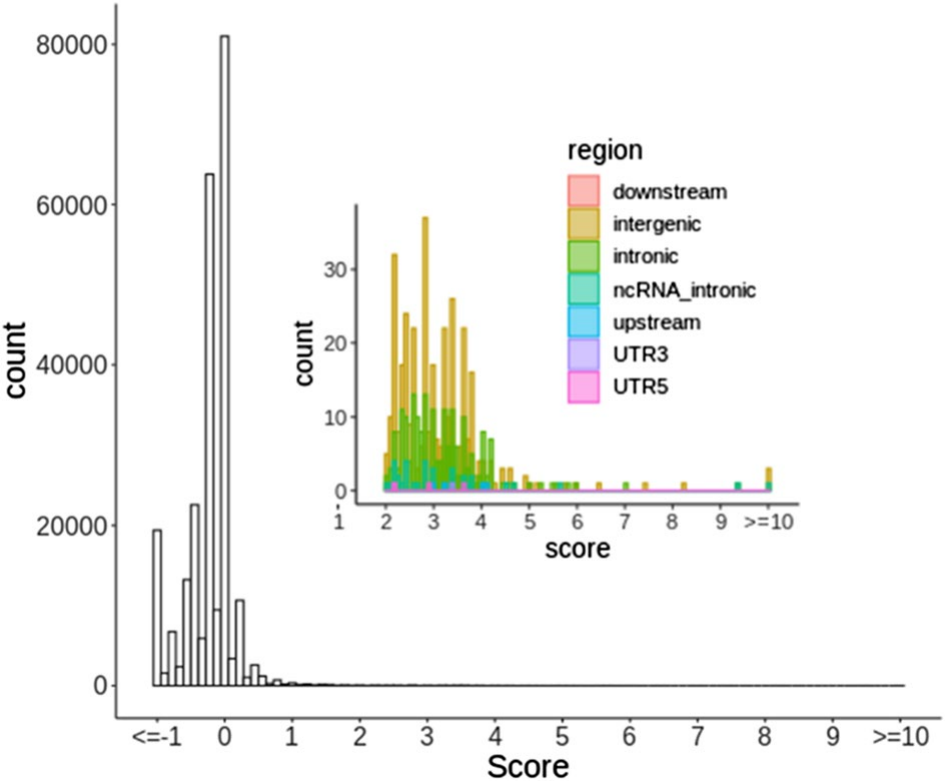
The distribution of repeat scores of whole-genome STRs in HG02059. The repeats with score ≥ 2 are annotated by wANNOVAR [41] to assess their functional impacts

We next demonstrate how to use RepeatHMM-scan and RepeatHMM-DB for whole-genome STR analysis. To do that, we randomly choose a genome (HG02059) from the 21 long-read data, and infer normal repeat ranges of whole genome STRs on the remaining 20 long-read datasets. Please note that this analysis is solely for demonstration purpose, and no repeat expansion disorders are known for the human individuals from the 21 long-read datasets. In this analysis, we run RepeatHMM-scan on long-read sequencing data on HG02059, and calculate the scores for 400,046 STR based on the inferred normal repeat ranges from the 20 long-read datasets. To avoid the strong bias of the scores by small deviations, we set the minimum deviation to 5. Additionally, we also require that the STR score > 2.0, the detected repeat counts available in at least 10 of 20 genomes and we skip poly-A and poly-T repeats. As a result, we decrease the number of whole-genome STR repeats from 400,046 to 603 for further analysis, as shown in Fig. 4. That is, the majority of STRs are filtered out as expected.

We next run wANNOVAR [41] on the 603 repeats, and find 357 STRs in intergenic regions, 190 in intronic regions, 27 in non-coding RNA intronic regions, 5 in downstream regions, 4 in upstream regions, 3 in UTR 5′ and 2 in UTR 3′, as shown in Fig. 4. Since none of them are in coding regions, we use the score threshold > 10.0, as non-coding regions contain more variable STRs than coding regions (Fig. 2d) [42]. As a result, we find only 18 STRs left for further analysis with 0.004% false positive rate from 400,046 STRs. In particular, no coding STRs are identified with larger repeat counts. This case study demonstrates how to use RepeatHMM-DB in real-world settings when there is no prior knowledge on whether there is a repeat expansion in a genome, by focusing on a small subset of most likely expansions for downstream analysis and manual examination.

## Discussions

STRs are abundant throughout human genome, and specific repeat expansions may be associated with human diseases. Existing works have detected normal repeat ranges and minimum pathogenic repeats for tens of repeat loci using time-consuming and labor-intensive wet-lab techniques. The pathogenic status of repeat counts of a test individual can be easily detected by comparing them against normal repeat ranges or against the minimum pathogenic repeat counts, which is an efficient way to analyze tens of well-studied repeat loci. However, the information on normal repeat ranges for tens of thousands of STR loci are not available in population-scale data, creating a challenge for the inference of pathogenic alleles that are potentially associated with human diseases. This situation becomes much worse for whole-genome analysis from long-read sequencing data to identify potential disease-associated repeat loci, because there is no database on the normal ranges of repeat counts for all STRs and normal repeat ranges of different repeat loci can vary greatly.

Therefore, in this study, we use RepeatHMM-scan to build the first database (RepeatHMM-DB) of repeat counts for all STRs in human genome from long-read whole-genome sequencing data. That is, with the help of RepeatHMM-DB, a repeat count from documented repeat loci in GRCh38/hg38 can be evaluated to see whether the repeat count is abnormal or not. For example, we evaluate repeat counts of CAG repeat in *ATXN3* to infer their pathogenic status and find that RepeatHMM-DB yields high accuracy in its inference. One can extend this study by using RepeatHMM-DB to check repeat loci whose prior knowledge of normal repeat ranges is not available. Additionally, a user can run RepeatHMM-scan to generate repeat counts of genome-wide STRs for a testing genome, and check whether abnormal repeat counts exist; abnormal repeat counts are excellent candidates for further analysis to identify disease-relevant repeat expansions. Furthermore, using the identical procedure, this study can also be extended on long-read data of other species. Thus, our tools are expected to facilitate the discovery of novel disease-relevant repeat expansions, when more and more long-read whole-genome sequencing datasets are available for disease variant discovery. Please note that our study is different from many existing works on repeat detection. In a repeat detection work, a tool is developed to detect repeat counts for a specific repeat loci or several repeat loci on sequencing data. In this study, we do not endeavor to improve the performance of repeat count estimation, but to build a framework for efficient and effective analysis for whole-genome STRs to pinpoint abnormal repeat counts for human disease studies.

There are several limitations which need to be overcome in the future. First, STR regions are downloaded from UCSC genome browser through the TRF (tandem repeat finder) [29] track and used as input of RepeatHMM directly. However, some of STRs do not have well-defined repeat motifs with either imperfect repeats or with > 1 available repeat motif for a complicated repeat region. We will overcome this issue in the future version of RepeatHMM-DB by using careful curation of STR regions. Second, RepeatHMM-DB is built with 21 long-read sequencing data (40 sets of alleles), so larger normal repeat counts that are rare may not be detected in these genomes. This issue can be addressed when more and more long-read sequencing data become available in future. Third, currently the repeat loci in RepeatHMM-DB is built on the GRCh38/hg38 coordinate, and we will also provide repeat loci information for the GRCh37/hg19 coordinate in future. Fourth, we use alignment files to infer repeat counts, and thus, the quality of alignments generated by different aligner might affect the results. Similarly, we used RepeatHMM-scan to generate RepeatHMM-DB, yet other repeat estimation tools that are available may have slightly different estimations of repeats than RepeatHMM-scan, which need to be considered when using RepeatHMM-DB for the discovery of abnormal repeat expansions. Finally, our tool and database may not handle repeats in highly complex genomic regions (such as sub-telomeric regions), as the repeat motifs and lengths in those regions in a reference genome cannot be reliably assayed even by long-read sequencing techniques.

## Conclusions

In conclusion, the extended RepeatHMM (RepeatHMM-scan) together with RepeatHMM-DB provides an effective way to detect potentially pathogenic repeat expansions at a genomic scale. With the wider application of long-read sequencing techniques in whole-genome sequencing studies, we expect that RepeatHMM-DB can speed up the discovery of pathogenic repeat expansions on undiagnosed diseases in the future.

## Data Availability

The extended RepeatHMM (RepeatHMM-scan and RepeatHMM-DB) and the results are available at https://github.com/WGLab/RepeatHMM.

## Abbreviations

RepeatHMM: Repeat detection by hidden Markov model
RepeatHMM-DB: The database of genome-wide normal repeats generated by RepeatHMM
RepeatHMM-scan: Genome scan module in RepeatHMM
STR: Short tandem repeat
SCA3: Spinocerebellar ataxia type 3
